# Real-world treatment patterns and outcomes among unresectable stage III non-small cell lung cancer

**DOI:** 10.1371/journal.pone.0314156

**Published:** 2024-11-25

**Authors:** Ashwini Arunachalam, Sneha Sura, John Murphy, Paul Conkling, Jerome Goldschmidt

**Affiliations:** 1 Merck & Co., Inc., Rahway, New Jersey, United States of America; 2 Ontada, Boston, Massachusetts, United States of America; 3 The US Oncology Network, Blacksburg, Virginia, United States of America; Duke University Medical Center: Duke University Hospital, UNITED STATES OF AMERICA

## Abstract

**Background:**

In 2018, the treatment options for unresectable stage III non-small cell lung cancer (NSCLC) changed with durvalumab, an immune checkpoint inhibitor (ICI), which was approved for consolidation therapy following concurrent chemoradiotherapy (cCRT) without disease progression. Despite durvalumab’s clinical benefit, many patients receiving this therapy developed progression. This study evaluated treatment patterns and clinical outcomes in real-world community oncology practices for patients with unresectable stage III NSCLC who received cCRT.

**Methods:**

This study used The US Oncology Network’s (iKnowMed) electronic health record database supplemented by chart review and included adults diagnosed with unresectable stage III NSCLC initiating cCRT between 11/01/2017 and 10/31/2019, with follow-up through 04/30/2022. cCRT included concurrent treatment with platinum-based chemotherapy and radiation therapy (+/-14 days). Real-world overall survival (rwOS) and real-world progression-free survival (rwPFS) were estimated from cCRT initiation using the Kaplan–Meier method.

**Results:**

Among 426 patients, 61.5% received durvalumab post-cCRT (cCRT+durvalumab) and 38.5% did not (cCRT alone). Death (28.3%) and disease progression (22.2%) were the most common reasons for not initiating durvalumab. The median age for the cCRT+durvalumab and cCRT alone cohorts were 70 and 71 years, and 71.8% and 61.6% had Eastern Cooperative Oncology Group performance status of 0–1, respectively. 51.5% of cCRT+durvalumab discontinued durvalumab, primarily due to adverse events (35.8%) and disease progression (28.4%). Median rwOS was 50.2 (95% confidence interval [CI]:41.4, not reached) and 11.6 (95% CI:6.5,15.9) months for cCRT+durvalumab and cCRT alone, respectively. Median rwPFS was 28.5 (95% CI:23.3,36.4) months for cCRT+durvalumab and 6.3 (95% CI:4.3,9.3) months for cCRT alone, respectively. 23.7% (cCRT+durvalumab) and 26.2% (cCRT alone) received subsequent treatment, of which, 59.7% (cCRT+durvalumab) and 46.5% (cCRT alone) received ICI.

**Conclusion:**

Four out of ten patients did not receive consolidation durvalumab mainly due to disease progression. Even among patients who initiated durvalumab, many patients relapsed and were retreated with ICIs. These findings underscore the need to refine treatment strategies for better outcomes in stage III unresectable NSCLC.

## Introduction

Lung cancer is the leading cause of death in the United States [1], with approximately 127,070 deaths annually [2]. Non-small cell lung cancer (NSCLC) accounts for more than 80% of all lung cancer cases and approximately 20 to 35% of these patients present with Stage III, locally advanced disease, the majority of these patients have unresectable tumors [3]. The 5-year survival for patients diagnosed with stage IIIA, IIIB, and IIIC disease is 36–41%, 24–26%, and 12–13%, respectively [3, 4].

Historically, the standard of care (SoC) for patients with unresectable stage III NSCLC was platinum-based chemotherapy administered concurrently with radiotherapy (cCRT), followed by active surveillance [5]. While the intent of cCRT is curative, a considerable proportion of patients will relapse [6], with 50% developing distant metastases [7]. Moreover, prognosis remains poor, with a median progression-free survival (PFS) of approximately 8 months and a 5-year overall survival (OS) rate of 37% [5, 8].

In 2018, treatment landscape for unresectable stage III NSCLC changed with the introduction of durvalumab, an immune checkpoint inhibitor (ICI). The phase III trial comparing consolidation durvalumab to placebo among unresectable stage III patients who did not have disease progression after two or more cycles of platinum-based chemoradiotherapy (PACIFIC trial), demonstrated significant improvement in PFS (16.8 months vs. 5.6 months) and OS (23.2 months vs. 14.6 months), leading to FDA approval in this clinical setting [9]. Consolidation treatment with one year durvalumab post-cCRT has become the standard of care [10], with a manageable safety profile [10].

Despite the survival benefit, only 1/3 of patients receiving consolidation durvalumab were alive and free of disease progression at 5 years [11, 12]. Real-world studies have also shown that nearly half of the patients experienced disease progression [13] and 43% patients received subsequent treatment after completion of durvalumab [14]. Moreover, not all patients who undergo cCRT are eligible for consolidation durvalumab due to residual toxicity, impaired performance status, or disease progression [15, 16]. Limited evidence exists regarding the real-world use of consolidation durvalumab among patients with unresectable stage III NSCLC. Therefore, this study investigated treatment utilization patterns and clinical outcomes in real-world community oncology practices for patients with unresectable stage III NSCLC who initiated cCRT. The findings of this study will provide insight into the unmet medical needs in this population.

## Methods

### Data source

The data was obtained from the electronic health record (EHR) system of The US Oncology Network, iKnowMed (iKM). The US Oncology Network comprises 1400 affiliated physicians operating in over 450 sites of care in different states, providing treatment to approximately 1 million US cancer patients annually. iKM is an integrated web-based, oncology-specific EHR and captures outpatient medical oncology practice encounter histories for patients in community-based care. These include patient demographics (e.g., age, race and gender), clinical (e.g., smoking status, disease diagnosis, diagnosis stages, performance status information, laboratory testing results) and treatment information (e.g., chemotherapy regimens, line of therapy and treatment dates) within the US Oncology Network.

Some key variables were anticipated to be poorly documented in the structured EHR fields (e.g., biomarkers) or only available in unstructured fields (e.g., reasons for treatment initiation, treatment discontinuation, reasons for not initiating durvalumab, radiation therapy, tumor assessment). To obtain these data, trained oncology professionals manually abstracted the information via chart reviews, capturing information as it was explicitly documented in the medical record. Sources of vital status (death) of patients included the Limited Access Death Master File (LADMF) from the Social Security Administration, publicly available obituaries, and death dates recorded in the EHR and chart review.

Approval from the Institutional Review Board (IRB) and Compliance/Privacy was obtained before starting the retrospective research. As the study involved analyzing existing data and records, the information was processed in a way that ensured research participants could not be directly identified. Patient informed consent was not required due to the nature of the study design. Therefore, the study protocol was granted an exception and waiver of informed consent by The US Oncology, Inc. IRB. All data were handled in compliance with the Health Insurance Portability and Accountability Act and the Health Information Technology for Economic and Clinical Health Act.

### Study design and population

This retrospective observational study included adult patients (≥18 years) newly diagnosed with unresectable stage III NSCLC according to the American Joint Committee on Cancer versions 7.0 and 8.0 [17, 18]. Patients initiating cCRT between 01 November 2017 (to characterize durvalumab use after its FDA approval in May 2017) and 31 October 2019 (study identification period) were included and followed till 30 April 2022, with a minimum follow-up of 30 months (data cutoff date), or death, or lost to follow-up, whichever occurred first (study observation period). Unresectable stage III NSCLC was defined as patients diagnosed with stage III NSCLC with no prior history of surgical treatment or patients who were not eligible for surgery prior to initiating chemotherapy and radiation. cCRT included concurrent use of platinum-based chemotherapy and radiation therapy (defined as radiotherapy received +/-14 days of receipt of the first dose of chemotherapy). The date of initiation of cCRT after the confirmed diagnosis of unresectable stage III NSCLC was defined as the index date. Eligible patients were required to have at least 2 physician visits (defined as physical encounters with the practice, detected by vital signs records; second and third visits were observed after the index date to demonstrate a continuity of care) within the US Oncology Network during the study observation period. Patients were excluded if they were enrolled in interventional clinical trials or received treatment for other documented primary cancer diagnoses during the study observation period.

Patients were initially identified through inclusion and exclusion criteria available in structured fields of the EHR. Among these identified patients, a random sample was selected to undergo a targeted chart review to confirm their eligibility.

### Study variables

#### Patient characteristics

Demographics characteristics included age, sex, race and practice region. Clinical characteristics included smoking status, body mass index (BMI), Eastern Cooperative Oncology Group (ECOG) Performance Status (PS), stage using American Joint Commissioner of Cancer (AJCC) criteria version 7.0 and version 8.0, T and N component of TNM staging, histology, programmed death-ligand 1 (PD-L1), time from stage III diagnosis to cCRT initiation. Age and stage were captured at index date; sex and race were obtained from prior medical history (any time prior to index date); practice location, smoking status, BMI, T and N component of TNM staging, ECOG PS, and histology were collected at baseline (6 months prior to the index date); age and ECOG PS were also captured at initiation of durvalumab.

#### Treatment patterns

Treatment patterns examined concurrent chemotherapy regimen of cCRT, number of cCRT cycles, and number of durvalumab cycles (defined as the duration of durvalumab divided by 28 days). Additionally, the study also examined duration of cCRT use (in months; defined as time from initiation to discontinuation of cCRT), time from the last dose of cCRT to durvalumab initiation (in months; defined as interval between the last dose of cCRT to the initiation of durvalumab), reasons for not initiating durvalumab as explicitly documented in patients’ notes using a pre-specified list, duration of durvalumab use (in months; defined as time from initiation to discontinuation of durvalumab), reasons for durvalumab discontinuation, proportion with locoregional (LRR) or metastatic recurrence (defined as time from initiation of index date to the evidence of first recurrence), and proportion receiving subsequent systemic anticancer treatment after cCRT or durvalumab (defined as first systemic anticancer treatment patients received following cCRT or durvalumab regardless of disease progression).

#### Clinical outcomes

Observed follow-up (in months) was defined as the duration between index date and the last date of contact or death date or end of study observation period (data cut-off date), whichever occurred first. Theoretical follow-up (in months) was defined as the duration between index date and the end of the study observation period.

Real-world OS (rwOS) was defined as the duration between the index date and the date of death by any cause as documented in the LADMF and/or the iKM EHR database. Patients who did not die within the study observation period were censored on the end of the study observation period (data cut-off date: 30 April 2022) or the last visit date, whichever occurred first. The hierarchy in the priority order for determining death date was chart review, LADMF, and programmatic query of the iKM database.

rwPFS was defined as the duration between index date to the earliest date of disease progression or date of death due to any cause. Patients who did not die or did not progress were censored on the end of the study observation period (data cut-off date: 30 April 2022) or the last visit date available in the dataset, whichever occurred first. Progression was captured based on the provider’s assessment reported in the patient charts in a scan report or progress note.

### Statistical analysis

We conducted descriptive statistics to summarize demographic and clinical characteristics, treatment patterns, and duration of follow-up, overall and by treatment category. Treatment patterns and outcomes were presented descriptively by receipt of durvalumab and were categorized as cCRT followed by durvalumab, defined as consolidation durvalumab [cCRT+durvalumab], or cCRT alone. Continuous variables were described using mean, standard deviation (SD), median, and interquartile range (IQR). Categorical variables were described by patient counts and percentages. Time-to-event analyses such as rwOS and rwPFS were estimated using the Kaplan-Meier product limit method. We assessed median survival times with 95% confidence intervals (CIs) and survival probabilities (with 95% CIs) at 12 and 24 months. Additional analysis was conducted for a subset of patients initiating durvalumab (cCRT+durvalumab) using date of initiation of durvalumab as the index date to examine clinical characteristics, rwOS and rwPFS. All statistical analyses were performed using SAS 9.4.

## Results

### Study population

A total of 18,994 adult patients were identified as having a stage III NSCLC diagnosis, of which, after applying selection criteria, a total of 959 unresectable stage III NSCLC patients who initiated cCRT were identified (S1 Fig). Of these, a random subset of 540 patients underwent detailed chart review, resulting in 426 patients meeting the eligibility criteria for the final analysis. Within this group, 262 (61.5%) received durvalumab following cCRT (cCRT+durvalumab) and 164 (38.5%) patients did not receive durvalumab (cCRT alone) within the study period.

### Demographic and clinical characteristics

Baseline patient characteristics are presented in Table 1. The median age was 70.6 (IQR: 63.7, 76.3) years, with 55.4% male and 72.3% White. Majority of patients had stage IIIA (48.6%) followed by stage IIIB (42.7%) at index. Nearly 82.9% were smokers and 67.8% had ECOG PS of 0–1. The median duration of observed follow-up since the index date was 18.2 (IQR: 6.4, 34.4), 30.4 (IQR: 13.5, 38.0) and 6.0 (IQR: 2.6, 15.1) months for overall, cCRT+durvalumab and cCRT alone cohort respectively. The median age of the cCRT+durvalumab cohort was 70.3 (IQR: 63.5, 75.9) years and that of the cCRT alone cohort was 71.2 (IQR: 63.9, 76.8) years. ECOG PS of 0–1 was more common in the cCRT+durvalumab cohort (71.8% vs 61.6% in cCRT alone cohort).

**Table 1:** Patient demographic and clinical characteristics of study population.

Characteristics	Overall (n = 426)	cCRT+durva (n = 262)	cCRT alone (n = 164)
**Age, median (IQR), years**	70.6 (63.7, 76.3)	70.3 (63.5, 75.9)	71.2 (63.9, 76.8)
**Sex (n, %)**			
Female	190 (44.6)	124 (47.3)	66 (40.2)
Male	236 (55.4)	138 (52.7)	98 (59.8)
**Race (n, %)**			
White	308 (72.3)	184 (70.2)	124 (75.6)
Others*	41 (9.6)	29 (11.0)	12 (7.3)
Not documented	77 (18.1)	49 (18.7)	28 (17.1)
**Practice region (n, %)**			
South	165 (38.7)	93 (35.5)	72 (43.9)
West	110 (25.8)	69 (26.3)	41 (25.0)
Midwest	127 (29.8)	86 (32.8)	41 (25.0)
Northeast	24 (5.6)	14 (5.3)	10 (6.1)
**BMI, median (IQR), kg/m** ^**2**^	25.4 (22.3, 28.7)	25.7 (22.2, 29.4)	24.7 (22.3, 28.1)
**Smoking status (n, %)**			
Current	109 (25.6)	62 (23.7)	47 (28.7)
Former	244 (57.3)	156 (59.5)	88 (53.7)
Never	23 (5.4)	14 (5.3)	9 (5.5)
Not documented	50 (11.7)	30 (11.5)	20 (12.2)
**Stage**^α^ **(n, %)**			
IIIA	207 (48.6)	135 (51.5)	72 (43.9)
IIIB	182 (42.7)	106 (40.5)	76 (46.3)
IIIC	37 (8.7)	21 (8.0)	16 (9.8)
**T component of TNM staging (n, %)**			
T0	<5	<5	0
T1	75 (17.6)	51 (19.5)	24 (14.6)
T2	80 (18.8)	52 (19.8)	28 (17.1)
T3	88 (20.7)	55 (21.0)	33 (20.1)
T4	115 (27.0)	71 (27.1)	44 (26.8)
TX	<5	<5	<5
Not documented	64 (15.0)	30 (11.5)	34 (20.7)
**N component of TNM staging (n, %)**			
N0	34 (8.0)	23 (8.8)	11 (6.7)
N1	37 (8.7)	19 (7.3)	18 (11.0)
N2	253 (59.4)	163 (62.2)	90 (54.9)
N3	94 (22.1)	55 (21.0)	39 (23.8)
NX	<5	<5	<5
Not documented	<5	<5	<5
**Histology (n, %**)			
Squamous cell	201 (47.2)	116 (44.3)	85 (51.8)
Non-squamous cell	225 (52.8)	146 (55.7)	79 (48.2)
**ECOG PS (n, %**)			
0–1	289 (67.8)	188 (71.8)	101 (61.6)
> = 2	55 (12.9)	27 (10.3)	28 (17.1)
Not documented	82 (19.3)	47 (17.9)	35 (21.3)
**PD-L1 (n, %)**			
Patients tested	230 (54.0)	150 (57.3)	80 (48.8)
<1%	70 (30.4)	41 (27.3)	29 (36.3)
1–49%	84 (36.5)	56 (37.3)	28 (35.0)
> = 50%	76 (33.0)	53 (35.3)	23 (28.8)
**Observed follow-up from cCRT initiation, median (IQR), months** ^#^	18.2 (6.4, 34.4)	30.4 (13.5, 38.0)	6.0 (2.6, 15.1)
**Theoretical follow-up from cCRT initiation, median (IQR), months** ^^^	41.1 (36.1, 46.7)	40.8 (35.6, 46.4)	41.8 (37.0, 47.4)
**Time from Stage III diagnosis to cCRT initiation, median (IQR), months**	1.2 (0.9, 1.7)	1.2 (0.8, 1.6)	1.3 (0.8, 1.8)

**Abbreviation:** BMI, Body mass index; cCRT, Concurrent chemoradiation therapy; Durva, Durvalumab; ECOG PS, Eastern Cooperative Oncology Group Performance Status; IQR, interquartile range; PD-L1, Programmed death-ligand 1

* Other races included African Americans, Asians, and Native Americans

^α^ Staging included AJCC v7.0 & 8.0

^#^ Observed follow-up (in months) was defined as the time between cCRT initiation date and the last date of contact/death/end of the study observation period (data cut-off date), whichever was the earliest

^^^ Theoretical follow-up (in months) was defined as the time between cCRT initiation date and the end of the study observation period (data cut-off date), whichever was the earliest

### Treatment patterns

Carboplatin plus paclitaxel (88.3%) was the most common chemotherapy regimens of cCRT among the overall study population, as well as among the cCRT+durvalumab (86.6%) and cCRT alone (90.9%) cohorts. In the cCRT+durvalumab cohort, the median duration of durvalumab treatment was 9.0 (IQR: 2.8, 11.8) months with a median number of cycles of 10 (IQR: 3, 13). The median time from the last dose of cCRT to durvalumab initiation was 1.6 (IQR: 1.2, 2.4) months (Table 2).

**Table 2:** Treatment patterns of study population.

Treatment patterns	Overall (n = 426)	cCRT+durva (n = 262)	cCRT alone (n = 164)
**Concurrent chemotherapy regimen (n, %)**			
Carboplatin+paclitaxel	376 (88.3)	227 (86.6)	149 (90.9)
Cisplatin+etoposide	36 (8.5)	26 (9.9)	10 (6.1)
Other*	14 (3.2)	9 (3.4)	5 (3.1)
**Time from the last dose of cCRT to durvalumab initiation, median (IQR), months**	-	1.6 (1.2, 2.4)	-
**Duration of cCRT, median (IQR), months**	1.4 (1.2, 1.5)	1.4 (1.2, 1.5)	1.3 (1.0, 1.5)
**Number of cCRT cycles, median (IQR)**	7.0 (7, 7)	7.0 (7, 7)	7.0 (7, 7)
**Duration of durvalumab, median (IQR), months** **	-	9.0 (2.8, 11.8)	-
**Number of durvalumab cycles, median (IQR)** **	-	10 (3, 13)	-
**Patient with evidence of disease progression (n, %)**	160 (37.6)	109 (41.6)	51 (31.1)
LRR only	34 (8.0)	24 (9.2)	10 (6.1)
Metastatic recurrence only	119 (27.9)	79 (30.2)	41 (25.0)
LRR followed by metastatic recurrence	7 (1.6)	6 (2.3)	-
**Patients receiving subsequent systemic anticancer treatments, (n, %)**	105 (24.6)	62 (23.7)	43 (26.2)
Chemotherapy combination	34 (32.4)	14 (22.6)	20 (46.5)
ICI monotherapy	29 (27.7)	14 (22.6)	15 (34.9)
ICI combination^#^	23 (21.9)	23 (37.1)	-
Others	19 (18.1)	11 (17.7) ^^^	8 (18.6) ***

**Abbreviation:** cCRT, Concurrent chemoradiation therapy; Durva, Durvalumab; ICI, Immune checkpoint inhibitor; IQR, interquartile range; LRR, Locoregional recurrence; SD, Standard deviation

* Other regimens of cCRT included cisplatin+pemetrexed, paclitaxel, carboplatin+pemetrexed, alectinib+etoposide

**One patient was excluded from the analysis of durvalumab duration and number of cycles due to being on durvalumab for 30 months

^#^ ICI combination treatments included ICI in combination with platinum or non-platinum.

^^^ Other treatments include non-platinum and targeted therapy

***Other treatments include ICI combination, non-platinum, and platinum. ICI combination was combined with others in cCRT alone cohort because of sample size <5

Overall, 37.6% had evidence of disease progression (LRR or metastatic recurrence) with 41.6% in the cCRT+durvalumab cohort and 31.1% in cCRT alone cohort. In the cCRT+durvalumab and cCRT alone cohorts, respectively, 23.7% and 26.2% received the first subsequent systemic anticancer treatment. In the cCRT+durvalumab cohort, majority of patients received ICI in combination (37.1%), ICI monotherapy (22.6%) or chemotherapy combination (22.6%). Similarly, the top two subsequent systemic anticancer treatments in the cCRT alone cohort are chemotherapy combination (46.5%) and ICI monotherapy (34.9%) **(**Table 2).

### Reasons for not initiating consolidation durvalumab

**Fig 1:**
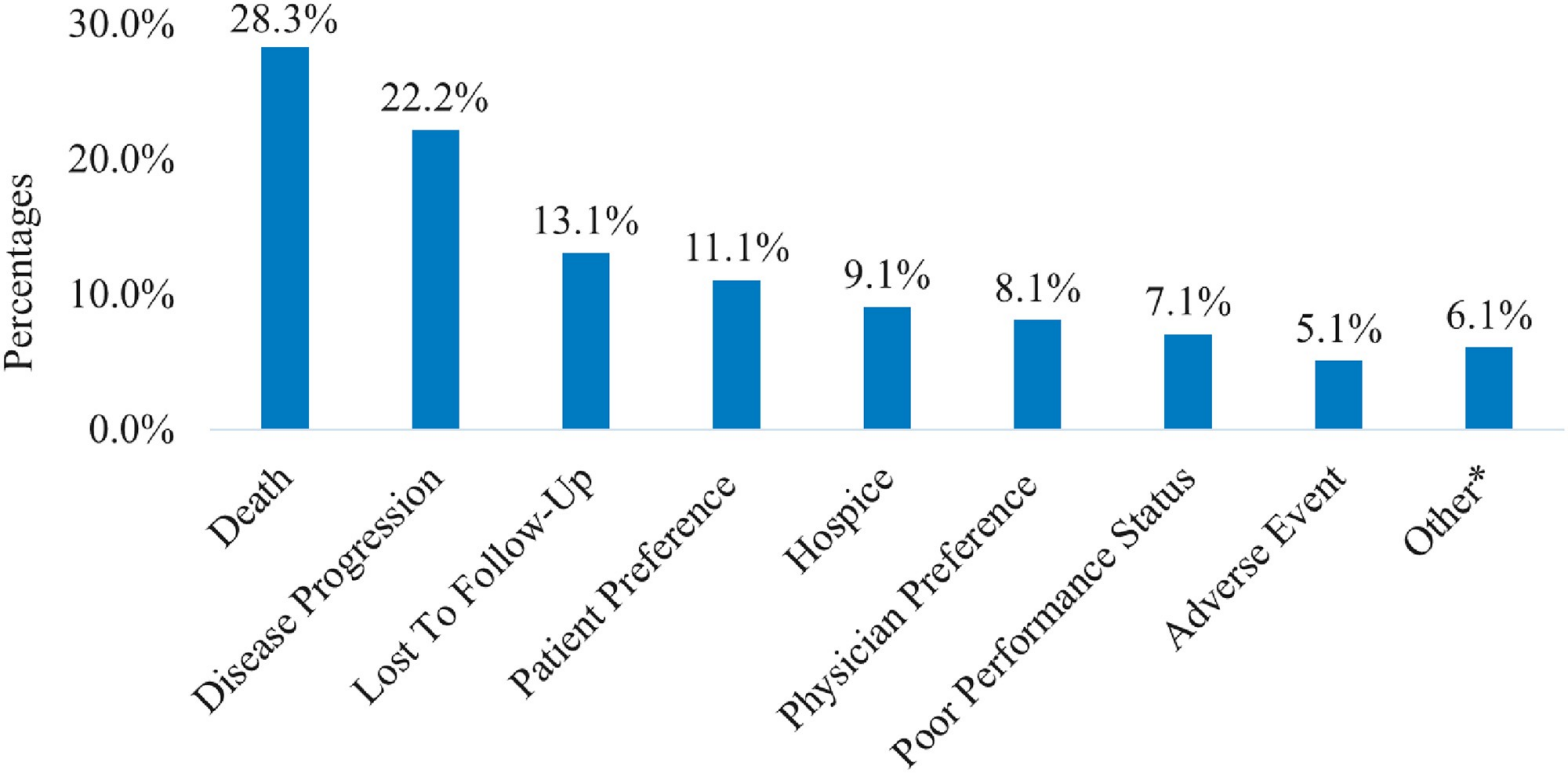
Reasons for not initiating durvalumab among the cCRT alone cohort. Percentages were calculated among patients with a documented responses and did not add up to 100% as patients could have more than one reason. Reasons for not initiating durvalumab were not documented among 39.6% of patients. *Other reasons included comorbidities, history of multiple sclerosis, poor tolerance to chemotherapy, financial/insurance, and stopped due to vision changes and dizziness.

Among patients with a documented response, the most common reported reasons for not initiating consolidation durvalumab among the cCRT alone cohort (n = 164) were death (28.3%), progression (22.2%), lost to follow-up (13.1%) and patient preference (11.1%) (Fig 1).

### Reasons for durvalumab discontinuation

**Fig 2:**
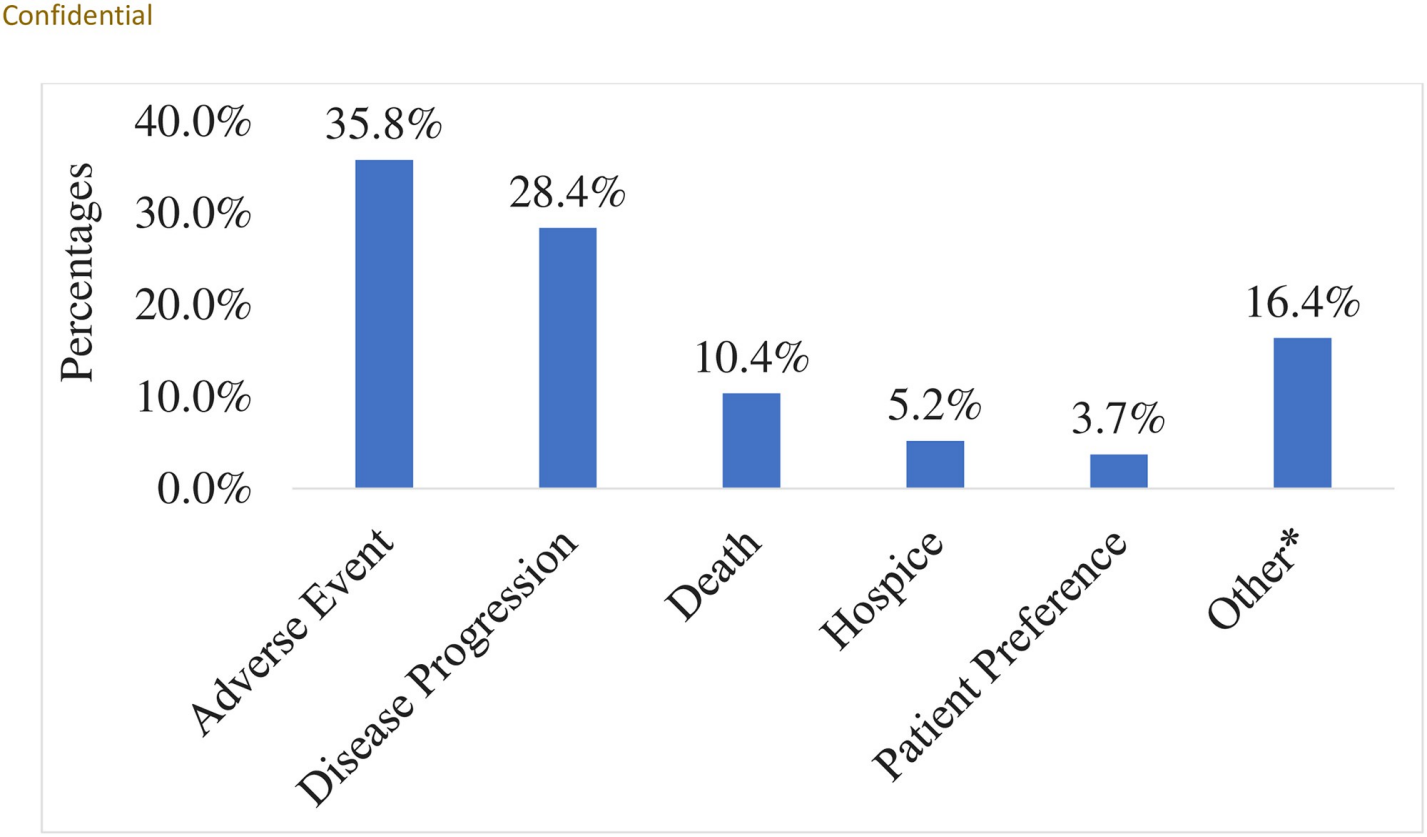
Reasons for durvalumab discontinuation. Reasons for durvalumab discontinuation are mutually exclusive. Therefore, percentages will add up to 100%. Percentages were calculated among patients with documented responses. Reasons for durvalumab discontinuation were not documented among 0.7% of patients. *Other reasons included poor performance status (PS), financial/insurance, progression & hospice, adverse event (AE) & patient preference, AE & poor PS, AE & hospitalization, death & hospice, AE & physician preference, death & hospitalization, poor PS & hospice, disease progression & death, and disease progression & death & hospice.

Among the cCRT+durvalumab cohort (n = 262), 48.1% of patients completed one-year of durvalumab treatment by the end of the study observation period, 51.5% discontinued durvalumab early, and there was one patient still on treatment at the end of the study observation period (0.4%). The most common reasons (mutually exclusive) for durvalumab discontinuation were adverse events (35.8%), disease progression (28.4%) and death (10.4%) (Fig 2).

### Clinical outcomes

#### Real-world overall survival (rwOS)

**Fig 3:**
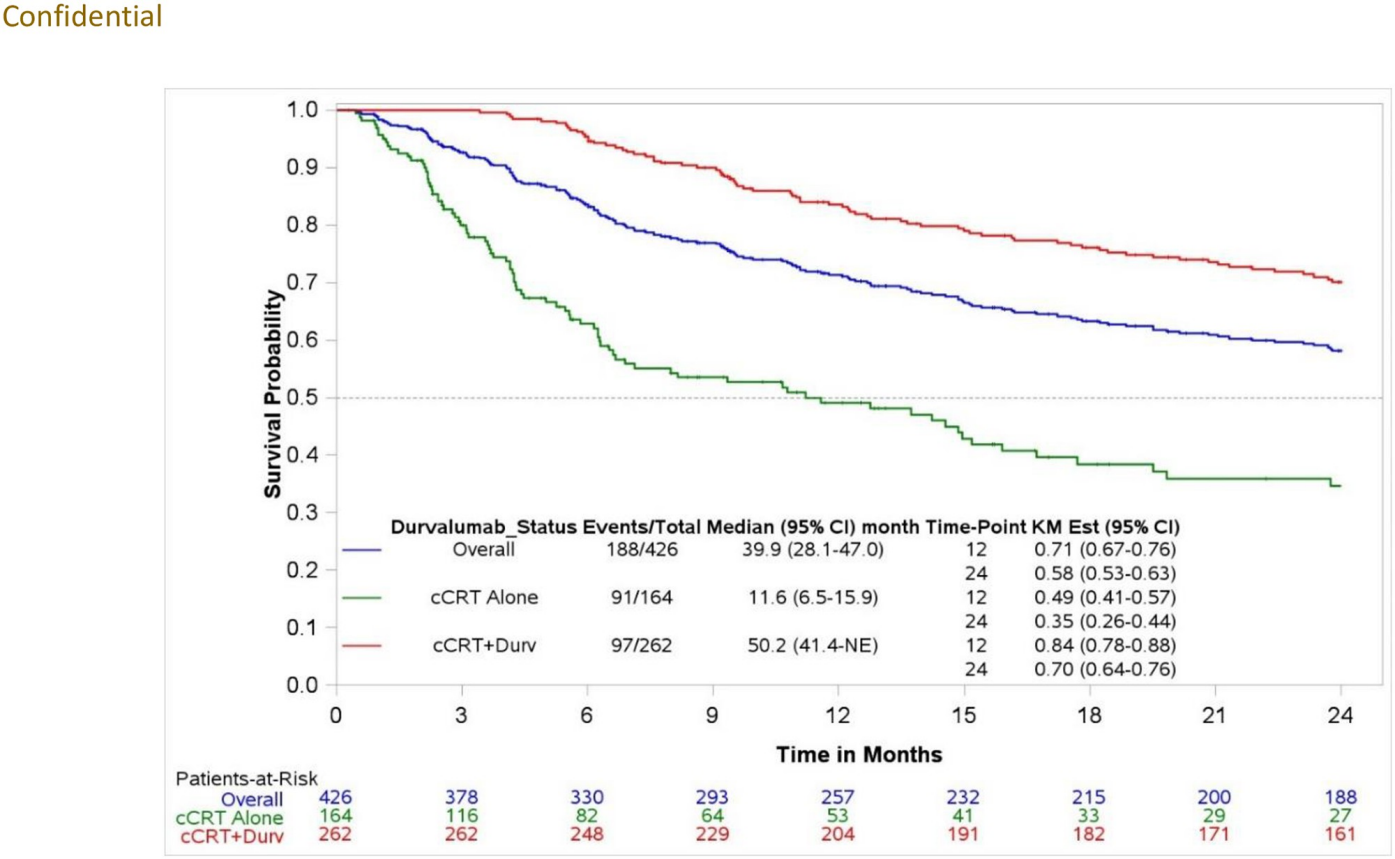
Real-world overall survival (rwOS) following cCRT initiation among the study population, stratified by the cCRT+durvalumab vs cCRT alone cohorts.

The median rwOS for the overall study population from index was 39.9 (95% CI: 28.1, 47.0) months, with an estimated 12-month survival rate of 71.3% (95% CI: 66.6, 75.5). Patients who received durvalumab post-cCRT had a median rwOS of 50.2 (95% CI: 41.4, not reached [NR]) months from index and a 12-month survival rate of 83.6% (95% CI: 78.4, 87.6). Patients initiating cCRT alone had a median rwOS of 11.6 (95% CI: 6.5, 15.9) months from index and a 12-month survival rate of 49.1% (95% CI: 40.4, 57.2) (Fig 3). Among the cCRT+durvalumab cohort, the median rwOS from consolidation durvalumab initiation was 46.6 (95% CI: 38.4, NR) months with a 12-month survival rate of 78.2% (95% CI: 72.5, 82.8) (S2 Fig).

#### Real-world progression-free survival (rwPFS)

**Fig 4:**
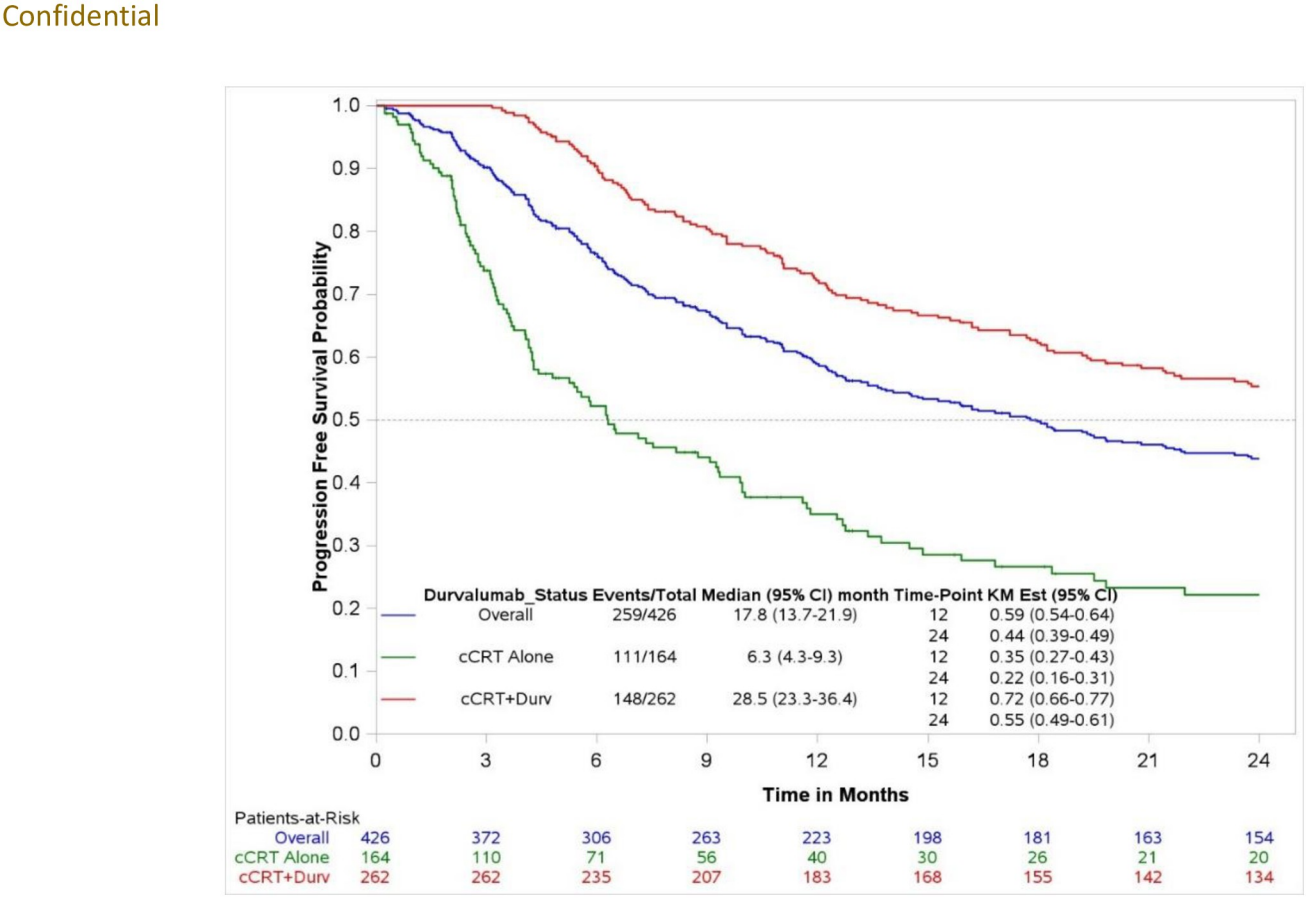
Real-world progression-free survival (rwPFS) following cCRT initiation among study population, stratified by the cCRT+durvalumab vs cCRT alone cohorts.

Overall, 259 (60.8%) patients showed evidence of disease progression or death following cCRT during the study observation period. The median rwPFS for the overall study cohort was 17.8 (95% CI: 13.7, 21.9) months with a 12-month rwPFS probability of 58.8% (95% CI: 53.8, 63.5). Among the durvalumab post-cCRT cohort, 56.5% developed progressive disease or died versus 67.7% in the cCRT alone cohort. Patients who received durvalumab post-cCRT had a median rwPFS of 28.5 (95% CI: 23.3, 36.4) months starting from index date and a 12-month rwPFS probability of 72.2% (95% CI: 66.3, 77.2). Patients initiating cCRT alone had a median rwPFS of 6.3 (95% CI: 4.3, 9.3) months and a 12-month rwPFS probability of 35.0% (95% CI: 27.1, 43.0) (Fig 4). Among the cCRT+durvalumab cohort, the median rwPFS from consolidation durvalumab initiation was 25.4 (95% CI: 20.7, 32.7) months, with a 12-month rwPFS probability of 65.8% (95% CI: 59.7, 71.3) (S3 Fig).

## Discussion

This study provides several key findings on the real-world treatment patterns and clinical outcomes in patients with unresectable stage III NSCLC treated with cCRT in a community oncology setting: 1) only 6 out of 10 patients received consolidation durvalumab; 2) the most common reasons for not receiving consolidation durvalumab were death (28.3%) and disease progression (22.2%); 3) more than half of the cCRT+durvalumab cohort discontinued durvalumab before completing all planned therapy, mainly due to adverse events (35.8%) and disease progression (28.4%); 4) one-fourth of cCRT+durvalumab cohort received subsequent systemic anticancer treatment. Despite receiving consolidation durvalumab post-cCRT, approximately 41.6% had disease progression; 5) most patients who developed disease progression after receipt of durvalumab are retreated with ICI therapy.

The potential underutilization of durvalumab in this real-world study can be attributed to death (28.3%), disease progression (22.2%) or patient preference (11.1%) after initial cCRT treatment. Disease progression was one of the most common reasons for not initiating durvalumab in several other real-world observational retrospective studies, observed among 9%-42% of the study populations [19–24]. Also, toxicity from CRT, comorbidities and poor performance status were other common reasons for not receiving durvalumab [19–24]. These findings suggest the need for more effective induction therapy for patients with unresectable Stage III NSCLC, to allow a greater number of these patients to have stable disease and continue consolidation treatment.

Consistent with prior real-world studies, we found that more than half of patients discontinued consolidation durvalumab, primarily due to adverse events (35.8%), disease progression (28.4%), or death (10.4%) [13, 19–23, 25, 26]. The findings from the PACIFIC clinical trial and PACIFIC-R study revealed that adverse events (15.4 vs. 16.9, respectively) and disease progression (26.9%) were the most prevalent causes for durvalumab discontinuation [10, 13]. Correspondingly, other real-world studies reported disease progression (ranging from 18% to 35%) and adverse events (ranging from 17% to 24%) as two of the most common reasons for discontinuation [13, 19–23, 25, 26]. The high discontinuation rates and adverse events in these studies suggest a need for different treatment strategies involving ICI that can help improve adherence and outcomes. Also, emerging evidence indicates that chemotherapy and radiotherapy can enhance the expression of PD-L1 in cells, making them more responsive to therapeutic agents that specifically target PD-L1 [27]. Numerous clinical trials are currently underway to explore innovative strategies for this patient population. These trials include concurrent ICI with cCRT and/or subsequent radiotherapy (KEYLYNK-012, EA5181, BRIDGE), the use of dual IOs after cCRT (COAST, PACIFIC-8, PACIFIC-9, SKYSCAPER-03, KEYVIBE-006), consolidation ICI following cCRT/subsequent CRT (PACIFIC-5, GEMSTONE 301), and ICI as an induction therapy preceding cCRT (APOLO) [28, 29].

Real-world clinical outcomes from this study were consistent with prior literature. The median duration of durvalumab (9.0 months) in this study aligns with other real-world world studies, which ranges from 8.5 to 9.0 months [24, 30]. This study’s median rwOS (46.6 months) for the cCRT+durvalumab cohort is consistent with the PACIFIC clinical trial (47.5 months) [10] but exceeded that of other real-world studies (around 34 months) [22, 25]. Median rwPFS from durvalumab initiation (25.4 months) in this study is better than the median PFS based on the PACIFIC clinical trial (16.8 months) [10] and other real-world studies (ranging from 16.9 months to 21.7 months) [13, 25]. Despite the clinical benefits of durvalumab, the 4 out of 10 patients who did not receive this treatment had dismal survival (median rwOS for cCRT cohort: 11.6 [95% CI: 6.5, 15.9]), suggesting an unmet treatment need in this patient population. This is a patient population with many comorbid illnesses who may not be able to withstand the rigors of cCRT. Patient selection is paramount among clinicians when deciding to prescribe cCRT in this population. Aside from performance status, there are neither predictive biomarkers nor clinical nomograms to help guide clinicians.

This study also revealed that 23.7% and 26.2% received subsequent systemic anticancer treatment among the cCRT+durvalumab and cCRT alone cohorts. Additionally, 41.6% and 51.1% experienced disease progression among the cCRT+durvalumab and cCRT alone cohorts, respectively. In both cohorts, ICI monotherapy or combination therapy were the most common subsequent systemic anticancer treatments. This is consistent with updated PACIFIC trial data [31] and real-world studies by Spigel et al. [12] and Planchard et al., [32] where 9.7%, 12.6%, and 4.2% of patients receiving durvalumab post-cCRT received ICI as subsequent treatment, respectively. This could be due to the reason that retreatments with ICI can restore or enhance the anti-tumor immune response by interrupting the signaling pathway of T-cell inhibition to help to positively regulate T-cell activity [33]. Although 14.1% of patients regardless of disease progression are getting retreated with ICIs as subsequent therapy, there is little clinical evidence for benefit in this patient population.

While durvalumab addresses a critical need for patients with unresectable stage III NSCLC, our study underscores the need for additional treatment strategies to address the limitations of consolidation treatment with ICI therapy and explore the best mode of application of ICI in this patient population. Few such strategies include concurrent ICI with cCRT, or induction ICI followed by cCRT. In both strategies, all patients eligible for cCRT would have an opportunity to receive ICI and may also exploit the potential synergism between chemotherapy and ICI. Notably, KEYNOTE-799 demonstrated a favorable response rate and safety profile associated with concurrent pembrolizumab with cCRT [34]. Currently, there are ongoing investigations into other ICIs for patients with unresectable locally advanced NSCLC (e.g., MK-7339-012/KEYLYNK-012, EA5181, MK-7684A-006/KEYVIBE-006) [29]. MK-7339-012/KEYLYNK-012 will compare pembrolizumab with cCRT followed by pembrolizumab or olaparib vs. cCRT alone followed by durvalumab in patients with unresectable locally advanced stage III NSCLC [35].

### Limitations

This observational and retrospective study uses iKM EHR data. The iKM database is not collected for research purposes but for clinical practice reasons. This may impede the standardization of the data collection methods and instruments and the reporting practices of the physician. As with all administrative databases, iKM data are subject to coding errors of omission and commission. Problems with inadequate or inaccurate codes in the databases may introduce some level of misclassification bias of certain diagnoses, events, or procedures of interest in the study. Likewise, some variables of interest may not be as complete across the entire population. The iKM EHR contains information on patients only when they are seen by The US Oncology Network clinics physicians. Services and procedures provided outside of The US Oncology Network are not captured by the database, as well as drugs received by patients from pharmacies not affiliated with The US Oncology Network practices. Additionally, the two treatment groups (cCRT+durvalumab and cCRT alone) were not randomized and could have differences in baseline characteristics leading to selection bias influencing clinical outcomes. Moreover, this study is descriptive in nature and did not compare treatment groups. We did not adjust for confounders or use statistical methods to impute missing data. Lastly, the OS estimates for the cCRT+durvalumab cohort should be interpreted with caution due to the potential immortal time bias as these patients were required to initiate durvalumab to be included in this subgroup.

## Conclusion

The findings highlight important aspects, including the underutilization of consolidation durvalumab, reasons for treatment discontinuation, recurrence rates, subsequent treatment rates, and clinical benefits. The study found nearly four out of ten unresectable stage III NSCLC patients did not receive the consolidation treatment with durvalumab. Despite the clinical benefits with consolidation durvalumab, a significant proportion of patients relapsed and were retreated with ICI agents. These findings have implications for future treatment strategies and highlight the need to improve clinical outcomes for this difficult to treat patient population.

## Supporting information

S1 Fig Patient flowchart. **Abbreviation**: NSCLC, non-small cell lung cancer; cCRT, concurrent chemoradiation therapy.
(TIF)

S2 Fig Kaplan-Meier plots depicting real-world overall survival (rwOS) following durvalumab initiation among overall cCRT+durvalumab patients. Abbreviations: cCRT, concurrent chemoradiation therapy.
(TIF)

S3 Fig Kaplan-Meier plots depicting real-world progression-free survival (rwPFS) following durvalumab initiation among overall cCRT+durvalumab patients. Abbreviations: cCRT, concurrent chemoradiation therapy.
(TIF)
