# Supplementary figures and images for: Real-world treatment patterns and outcomes among unresectable stage III non-small cell lung cancer
Source: PLoS One. 2024 Nov 25;19(11):e0314156. doi: 10.1371/journal.pone.0314156 (PMC11588217; doi:10.1371/journal.pone.0314156)

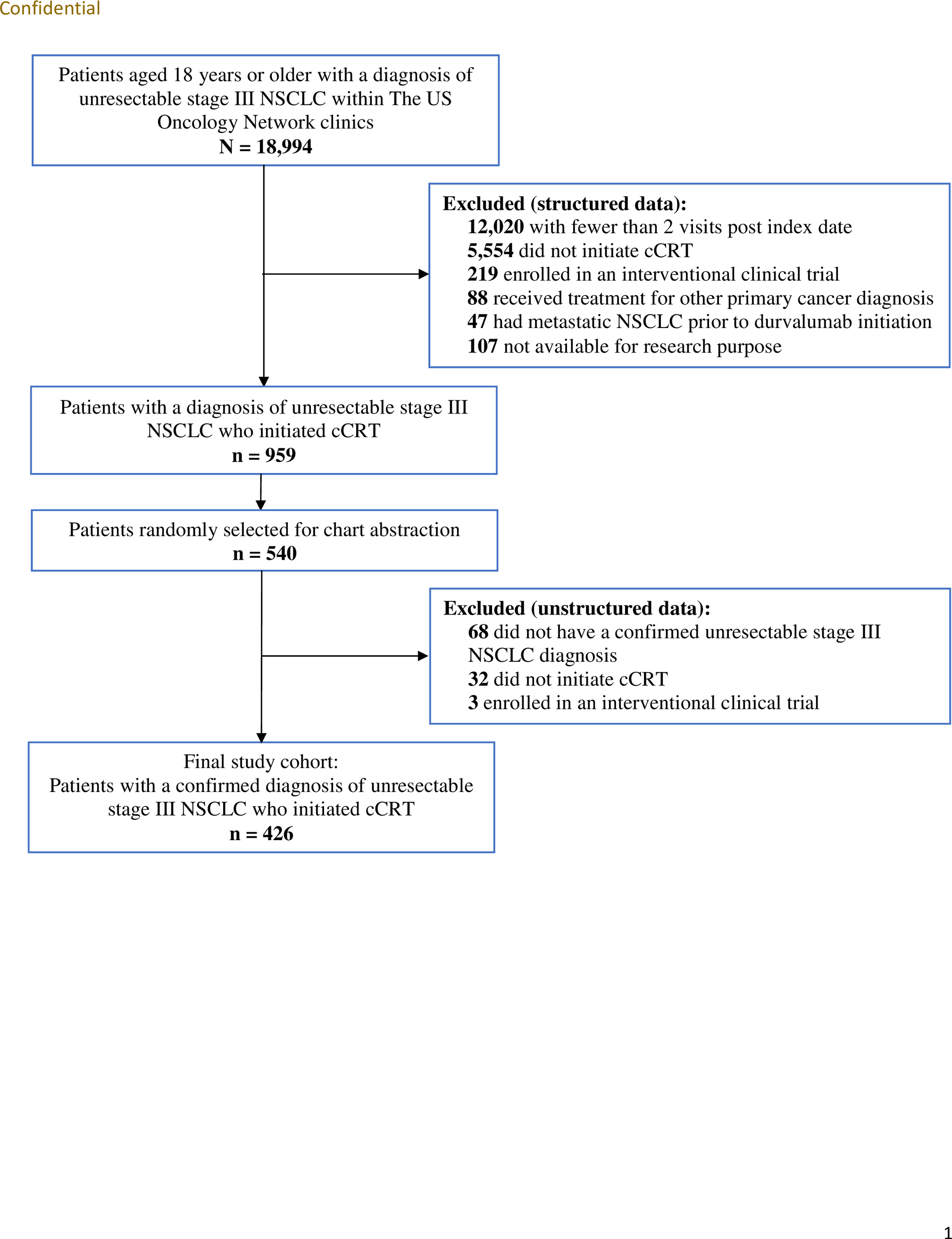

Supplement: S1 Fig — Abbreviation: NSCLC, non-small cell lung cancer; cCRT, concurrent chemoradiation therapy. (TIF) [file pone.0314156.s001.tif]

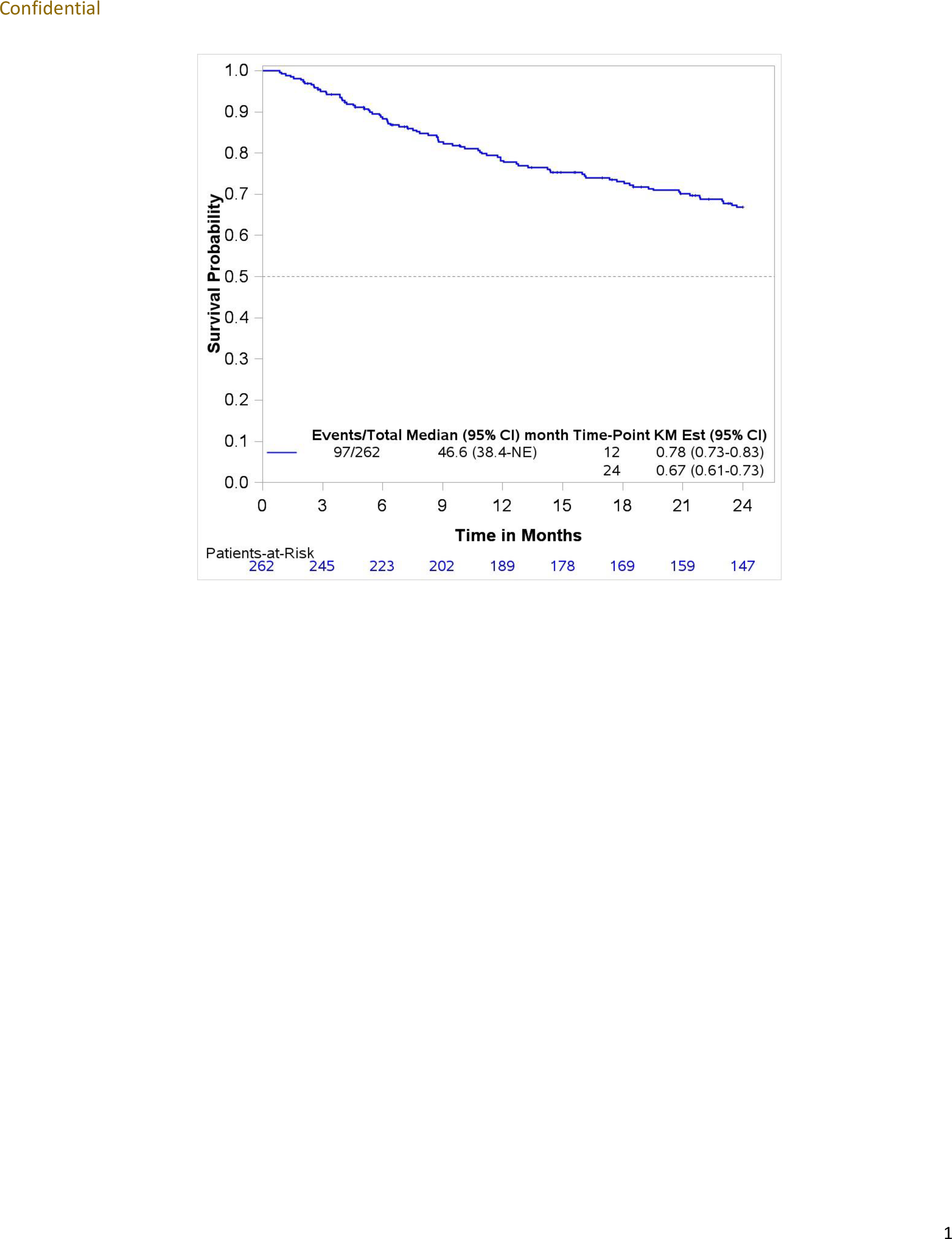

Supplement: S2 Fig — Abbreviations: cCRT, concurrent chemoradiation therapy. (TIF) [file pone.0314156.s002.tif]

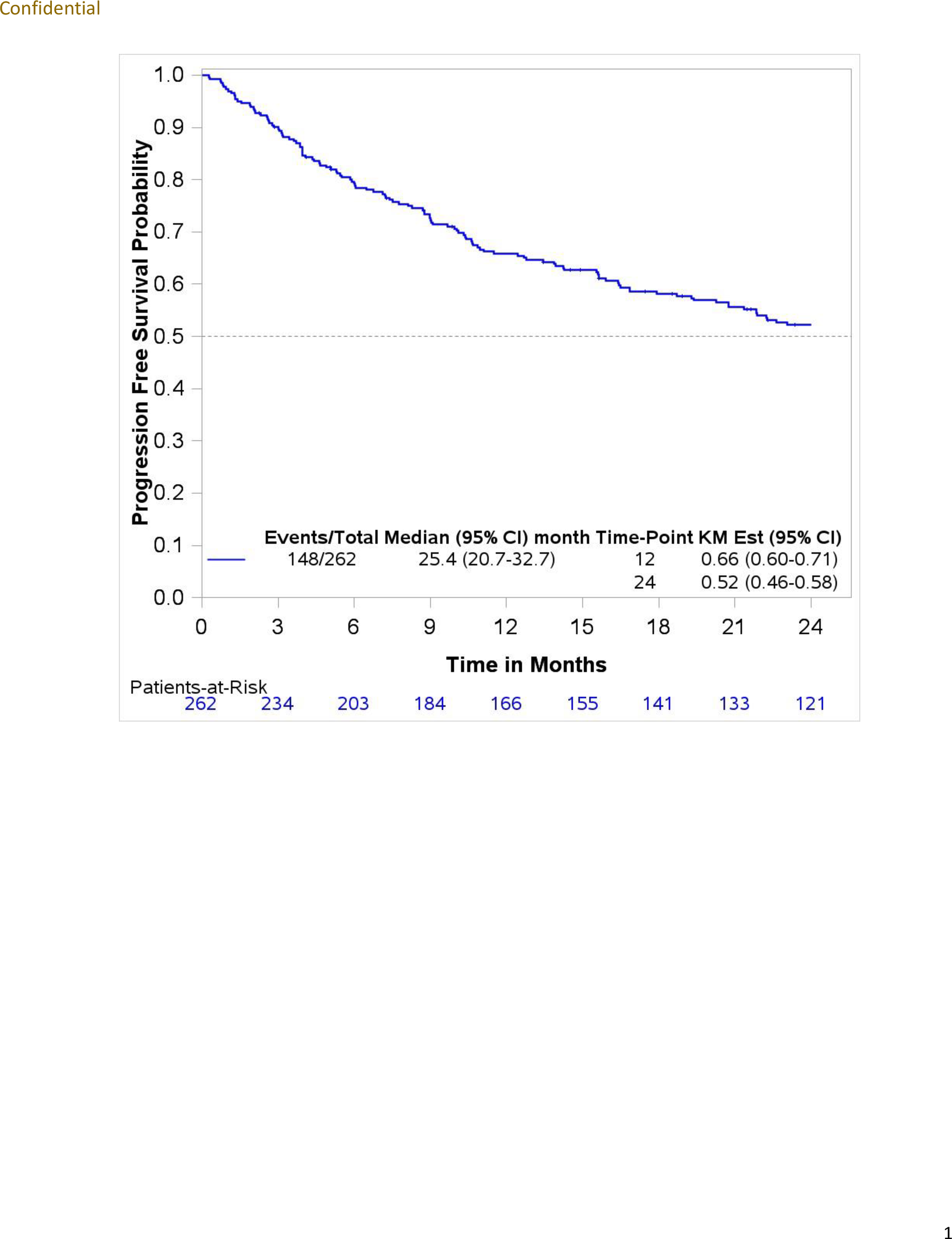

Supplement: S3 Fig — Abbreviations: cCRT, concurrent chemoradiation therapy. (TIF) [file pone.0314156.s003.tif]
